# Smooth trends in fermium charge radii and the impact of shell effects

**DOI:** 10.1038/s41586-024-08062-z

**Published:** 2024-10-30

**Authors:** Jessica Warbinek, Elisabeth Rickert, Sebastian Raeder, Thomas Albrecht-Schönzart, Brankica Andelic, Julian Auler, Benjamin Bally, Michael Bender, Sebastian Berndt, Michael Block, Alexandre Brizard, Pierre Chauveau, Bradley Cheal, Premaditya Chhetri, Arno Claessens, Antoine de Roubin, Charlie Devlin, Holger Dorrer, Christoph E. Düllmann, Julie Ezold, Rafael Ferrer, Vadim Gadelshin, Alyssa Gaiser, Francesca Giacoppo, Stephane Goriely, Manuel J. Gutiérrez, Ashley Harvey, Raphael Hasse, Reinhard Heinke, Fritz-Peter Heßberger, Stephane Hilaire, Magdalena Kaja, Oliver Kaleja, Tom Kieck, EunKang Kim, Nina Kneip, Ulli Köster, Sandro Kraemer, Mustapha Laatiaoui, Jeremy Lantis, Nathalie Lecesne, Andrea Tzeitel Loria Basto, Andrew Kishor Mistry, Christoph Mokry, Iain Moore, Tobias Murböck, Danny Münzberg, Witold Nazarewicz, Thorben Niemeyer, Steven Nothhelfer, Sophie Péru, Andrea Raggio, Paul-Gerhard Reinhard, Dennis Renisch, Emmanuel Rey-Herme, Jekabs Romans, Elisa Romero Romero, Jörg Runke, Wouter Ryssens, Hervé Savajols, Fabian Schneider, Joseph Sperling, Matou Stemmler, Dominik Studer, Petra Thörle-Pospiech, Norbert Trautmann, Mitzi Urquiza-González, Kenneth van Beek, Shelley Van Cleve, Piet Van Duppen, Marine Vandebrouck, Elise Verstraelen, Thomas Walther, Felix Weber, Klaus Wendt

**Affiliations:** 1 GSI Helmholtzzentrum für Schwerionenforschung, Darmstadt, Germany; 2 Department Chemie, Johannes Gutenberg-Universität Mainz, Mainz, Germany; 3 Helmholtz-Institut Mainz, Mainz, Germany; 4 Department of Chemistry, Nuclear Science & Engineering Center, Colorado School of Mines, Golden, CO USA; 5 University of Groningen, Groningen, The Netherlands; 6 ESNT, IRFU, CEA, Université Paris-Saclay, Gif-sur-Yvette, France; 7 Université Claude Bernard Lyon 1, CNRS/IN2P3, IP2I, UMR 5822, Villeurbanne, France; 8 GANIL, Caen, France; 9 University of Liverpool, Liverpool, UK; 10 Department of Physics and Astronomy, KU Leuven, Leuven, Belgium; 11 Oak Ridge National Laboratory, Oak Ridge, TN USA; 12 Institut für Physik, Johannes Gutenberg-Universität Mainz, Mainz, Germany; 13 Facility for Rare Isotope Beams, Michigan State University, East Lansing, MI USA; 14 Department of Chemistry, Michigan State University, East Lansing, MI USA; 15 Université Libre de Bruxelles, Brussels, Belgium; 16 CEA, DAM, DIF, Arpajon, France; 17 Université Paris-Saclay, CEA LMCE, Bruyères-le-Châtel, France; 18 Universität Greifswald, Greifswald, Germany; 19 Institut Laue-Langevin, Grenoble, France; 20 Helmholtz Forschungsakademie Hessen für FAIR (HFHF), GSI Helmholtzzentrum für Schwerionenforschung, Darmstadt, Germany; 21 TU Darmstadt, Darmstadt, Germany; 22 University of Jyväskylä, Jyväskylä, Finland; 23 Department of Physics and Astronomy, Michigan State University, East Lansing, MI USA; 24 Universität Erlangen, Erlangen, Germany; 25 IRFU, CEA, Université Paris-Saclay, Gif-sur-Yvette, France; 26 Division HÜBNER Photonics, HÜBNER, Kassel, Germany; 27 University of Gothenburg, Gothenburg, Sweden; 28 Present Address: Experimental Physics Department, CERN, Geneva, Switzerland

**Keywords:** Experimental nuclear physics, Superheavy elements

## Abstract

The quantum-mechanical nuclear-shell structure determines the stability and limits of the existence of the heaviest nuclides with large proton numbers *Z* ≳ 100 (refs. ^1–3^). Shell effects also affect the sizes and shapes of atomic nuclei, as shown by laser spectroscopy studies in lighter nuclides^4^. However, experimental information on the charge radii and the nuclear moments of the heavy actinide elements, which link the heaviest naturally abundant nuclides with artificially produced superheavy elements, is sparse^5^. Here we present laser spectroscopy measurements along the fermium (*Z* = 100) isotopic chain and an extension of data in the nobelium isotopic chain (*Z* = 102) across a key region. Multiple production schemes and different advanced techniques were applied to determine the isotope shifts in atomic transitions, from which changes in the nuclear mean-square charge radii were extracted. A range of nuclear models based on energy density functionals reproduce well the observed smooth evolution of the nuclear size. Both the remarkable consistency of model prediction and the similarity of predictions for different isotopes suggest a transition to a regime in which shell effects have a diminished effect on the size compared with lighter nuclei.

## Abstract (web summary)

Laser spectroscopy measurements of the fermium isotopic chain show a smooth trend in the nuclear size of heavy actinide elements, and diminishing shell effects on the size evolution compared with lighter nuclei.

## Main

The heaviest nuclei known so far owe their existence to quantum-mechanical nuclear-shell effects. These increase the stability of nuclei against spontaneous fission, enabling the formation of superheavy nuclei^1–3^. At specific numbers of protons (*Z*) or neutrons (*N*), so-called magic numbers, nucleonic shells show large energy gaps^6^, resulting in increased nuclear stability. This is analogous to the closed electron shells of noble gases resulting in their chemical inertness. The heaviest known nucleus with a magic number for both protons (*Z* = 82) and neutrons (*N* = 126) is ^208^Pb, a spherical nucleus. The location of the next spherical shell gap beyond ^208^Pb is yet unknown; nuclear models predict it most frequently at *Z* = 114, *Z* = 120 or *Z* = 126, and *N* = 172 or *N* = 184 (refs. ^2,7^). This variation in the predictions is primarily, among other factors, owing to a large single-particle level density in the heaviest nuclei^7^.

**Fig. 1:**
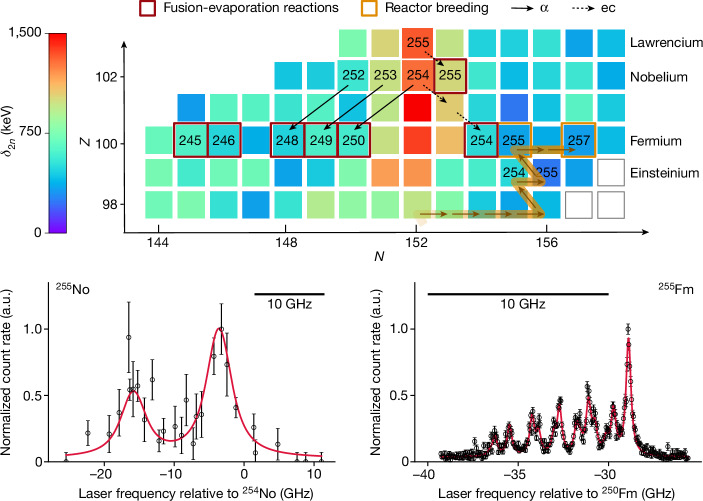
Overview of the investigated isotopes. Top: cut-out of the chart of nuclides in the heavy actinide region. The neutron shell gap parameter *δ*_2*n*_, calculated from experimental binding energies reported in ref. ^53^, as analogously deduced in ref. ^11^, is presented with colour coding. The isotopes studied in this work by laser spectroscopy are labelled, with nuclides studied on-line (red frames) and those studied off-line (orange frames). The black solid arrows indicate the α-decay path (α) and the dashed arrows indicate the electron-capture decay path (ec) utilized in the respective indirect production schemes. The orange arrows show the reactor-breeding path for fermium isotopes studied off-line (Methods). Bottom: on-line data on ^255^No (left) complemented by off-line data on ^255^Fm (right) with error bars showing statistical uncertainties (one standard deviation). The horizontal markers indicate a 10-GHz width. The red solid lines show fits to the data. a.u., arbitrary units. Source Data

Nuclei with proton numbers residing between magic numbers are expected to have deformed shapes owing to the nuclear Jahn–Teller effect^8,9^. The stabilization of deformed nuclei can be associated with the reduced density of the deformed single-particle levels of the nuclear mean field^6^. In the region of heavy nuclei beyond ^208^Pb, a deformed subshell at *N* = 152 was early identified through irregularities in the systematics of the α-decay energies of californium (*Z* = 98) isotopes, deviating from spherical shell model considerations^10^. Recently, precise mass measurements enabled a direct investigation of the *N* = 152 neutron shell gap in nobelium (No, *Z* = 102) and lawrencium (Lr, *Z* = 103) isotopes. The size of this subshell was determined from the experimental binding energies to be about a factor of four weaker than in ^208^Pb (refs. ^11–13^). As illustrated in Fig. 1 (top), the *N* = 152 gap gradually decreases in the lighter isotones. This result, consistent with spectroscopic studies^14^ and the recent analysis of experimental and theoretical binding energies^15^, confirms the local nature of this shell effect.

From in-beam γ-ray spectroscopy experiments, a large prolate deformation for the *N* = 152 nucleus ^252^Fm with a quadrupole deformation parameter *β* ≈ 0.3 was established from the observed rotational band structure, along with *K* isomers in this region^16–18^.

Nuclear charge radii, measured along a series of isotopes, offer another powerful probe of shell effects^19,20^, as they are sensitive to changes in the nuclear size and in nuclear deformation^21,22^. A prominent kink in the nuclear size evolution is often observed across spherical shell closures^23–26^. Here laser spectroscopy studies can be decisive in determining the trends in differential nuclear mean-square charge radii δ⟨*r*^2^⟩ along an isotopic chain by measuring isotope shifts in atomic transitions in a nuclear model-independent manner. Hyperfine splittings of atomic energy levels additionally give access to nuclear moments, providing insight into the nature of isomeric and ground-state nuclear configurations, for instance, shown in ref. ^27^. Such studies have already been showcased for many short-lived and rare lighter nuclides close to and far from stability^4,28^. In previous measurements applying laser spectroscopy around *N* = 32 in neutron-rich potassium isotopes^20^, the weak subshell closure was found to not manifest itself in the charge radii. In the region of nickel isotopes around the *N* = 40 subshell gap, a weak localized effect was identified in the charge radius evolution relative to the droplet-model trend^4,29^. This raises the question whether the *N* = 152 gap, which is reflected in nuclear binding energies, affects also the size evolution.

However, laser spectroscopy of the heaviest actinide elements and beyond is limited by production capabilities and sparse information on atomic energy levels^5^. Therefore, experimental information on the evolution of charge radii around deformed shell gaps in the heavy element region is scarce, and their description is challenging for nuclear models.

The development of the Radiation Detected Resonance Ionization Spectroscopy (RADRIS) method^30,31^ enabled such measurements, as demonstrated in laser spectroscopy of nobelium with atom-at-a-time production yields^32^. Isotope shifts in ^252,253,254^No up to *N* = 152 were investigated to deduce changes in ⟨*r*^2^⟩ and to obtain the electromagnetic moments of ^253^No. These experimental results are in good agreement with the predictions based on energy density functional (EDF) models, indicating a central depression by about 10% in the proton density distribution of ^254^No (ref. ^33^).

In this work, the combination of more recent advancements of the on-line (accelerator-based production) RADRIS method and highly sensitive off-line measurements enabled the determination of the isotope shift for an atomic transition in 8 fermium (Fm, *Z *= 100) isotopes across *N* = 152, probing the influence of the deformed *N* = 152 shell gap on ⟨*r*^2^⟩. Complementary to our fermium studies, we extended the isotope-shift measurements in nobelium isotopes with RADRIS beyond the shell gap, giving insight into the impact of the shell gap along another close lying isotopic chain.

The isotopes studied on-line were available through the following schemes: direct production of ^245,246^Fm in fusion-evaporation reactions, indirect production of ^248,249,250,254^Fm via the decay of directly produced ^252,253,254^No, and indirect production of ^255^No via the electron-capture decay branch of ^255^Lr. These indirect production schemes evolved from recent methodical advancements that gave access to previously inaccessible isotopes^34^ (for details, see Methods). However, the isotopes ^251−253^Fm are currently not accessible by this technique, not least owing to long half-lives of more than 5 hours up to several days.

Directly produced isotopes were separated from the primary beam by the recoil separator SHIP (Separator for Heavy Ion reaction Products) at GSI Helmholtzzentrum für Schwerionenforschung^35,36^ and transmitted to the gas-filled RADRIS stopping cell. Fusion-evaporation products were thermalized in the gas cell and collected and neutralized on a catcher filament. Pulse heating of the filament led to desorption of the atoms, followed by resonant laser ionization. The resulting ions were identified via their characteristic α-decay energy. This technique was recently improved for higher sensitivity^37^, which enabled laser resonance ionization spectroscopy with rates down to one particle every 100 seconds in the gas cell for ^246^Fm (Methods).

Off-line laser spectroscopy was accomplished at the RISIKO mass separator (Resonance Ionization Spectroscopy in Collinear geometry) on macroscopic sample sizes of neutron-rich fermium isotopes^38–40^. Reactor breeding of heavy actinides at the Oak Ridge National Laboratory’s High Flux Isotope Reactor produced samples of ^254^Es (half-life *t*_1/2_ = 275 d) and femtogram amounts of ^257^Fm (*t*_1/2_ = 100 d) (Fig. 1). The ^254^Es fraction was re-irradiated in the high-flux reactor at Institut Laue-Langevin, producing ^255^Es, which undergoes β^−^-decay leading to ^255^Fm. Every few days, fermium was chemically separated from einsteinium (Es, *Z *= 99), yielding these ^255^Fm samples to be used at RISIKO. Here fermium atoms were ionized in a hot-cavity laser ion source, and the resulting ions were accelerated and separated with a dipole magnet and then counted^41^ (Methods).

For studies in fermium, the known atomic transition from the 5*f*^12^7*s*^2^ ^3^H_6_ atomic ground state to the excited level 5*f*^12^7*s*7*p* $\genfrac{}{}{0ex}{}{5}{}{{\rm{G}}}_{5}^{{\rm{o}}}$ (refs. ^42,43^) was probed as the first excitation step in a two-step laser ionization scheme by registering ions from resonance ionization as a function of laser frequency detuning, as shown in Fig. 1 (bottom). Limited information on atomic levels in fermium was available from previous studies on ^255^Fm (refs. ^42,43^) and in nobelium from previous on-line studies with RADRIS^32^. Isotope shifts measured for the mentioned ground-state transition relative to the reference isotopes ^250^Fm and ^254^No, combined with input from atomic calculations^33,44^, allowed the extraction of changes in ⟨*r*^2^⟩ (Methods). The results are summarized in Table 1.

**Table 1:** Summary of laser resonance centroid wavenumbers (in argon buffer-gas environment), corresponding isotope shifts and evaluated changes in ⟨*r*^2^⟩

Fermium *Z* = 100			
*A*	Centroid (cm^−1)^	δ*ν*^250,*A*^ (GHz)	δ⟨*r*^2^⟩^250,*A*^ (fm^2^)
245	25 113.81(7)	30.4(2.2)	−0.323(24)[33]
246	25 113.50(5)	21.0(1.7)	−0.223(19)[23]
248	25 113.11(3)	9.5(1.0)	−0.101(10)[10]
249	25 112.99(11)	5.8(3.4)	−0.062(36)[6]
250	25 112.80(3)	0	0
254	25 111.90(4)	−27.0(1.4)	0.286(15)[29]
255	25 111.75(2)	−31.5(0.9)	0.335(10)[34]
257	25 111.48(8)	−39.5(2.4)	0.420(25)[42]

Nobelium *Z* = 102			
*A*	Centroid (cm^−1^)	δ*ν*^254,*A*^ (GHz)	δ⟨*r*^2^⟩^254,*A*^ (fm^2^)
255	29 961.20(3)	−7.7(0.8)	0.080(8)[6]

Isotope shifts are denoted by δ*ν*^250,*A*^ for measured shifts relative to ^250^Fm and δ*ν*^254,*A*^ for those measured relative to ^254^No. Statistical uncertainties (one standard deviation) are given in parentheses and systematic uncertainties are included in square brackets. The data analysis procedure is described in Methods.

**Fig. 2:**
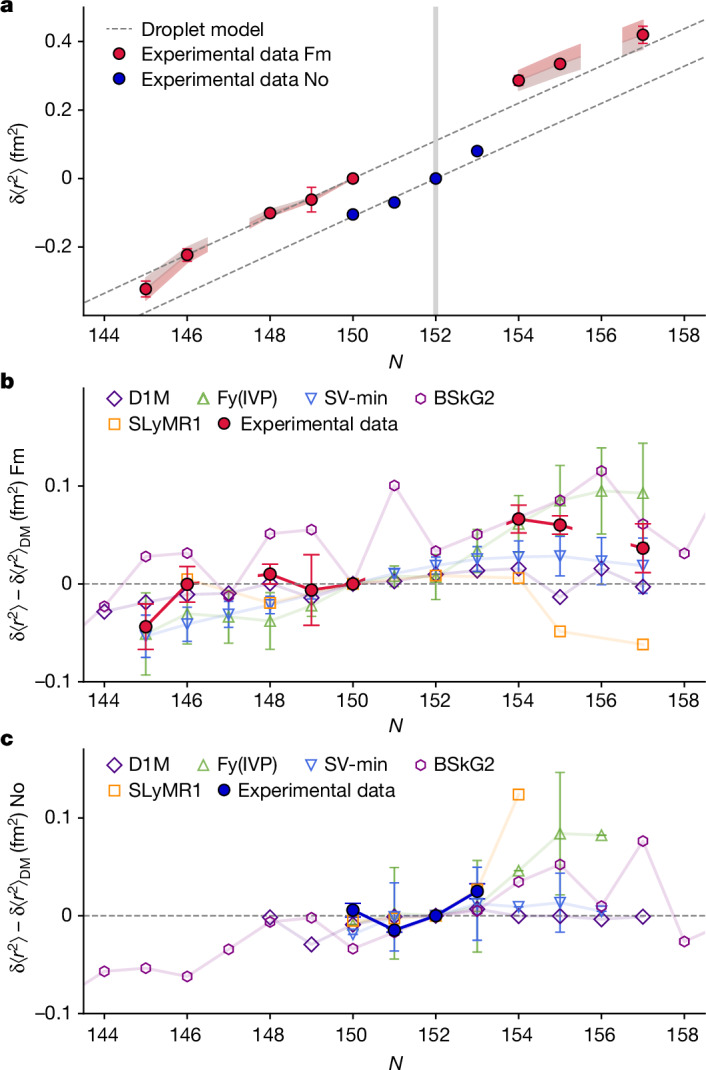
Comparison of experimental mean-square charge radii data with different nuclear model predictions. **a**, Experimental results of δ⟨*r*^2^⟩ of fermium isotopes as a function of *N* relative to ^250^Fm (red) and of nobelium isotopes relative to ^254^No (blue). The error bars show statistical uncertainties (one standard deviation) and the red shaded bands represent systematic uncertainties in the prediction of the atomic parameters (Methods). The observed smooth trend along the isotopic chain is independent of the atomic parameters as their uncertainty is only of a systematic nature, thus affecting all extracted δ⟨*r*^2^⟩ in the same manner. Predictions of the spherical droplet model are shown with dashed lines for comparison with the experimental data. **b**,**c**, δ⟨*r*^2^⟩ for fermium (**b**) and nobelium (**c**) predicted by five theoretical models relative to the droplet-model (DM) reference. Experimental data (red and blue solid symbols) are compared with predictions of different models. Source Data

To interpret the measured values, nuclear calculations were carried out using several EDF-based models, including Skyrme-type (SV-min, SLyMR1 and BSkG2), Gogny-type (D1M) and Fayans-type (Fy(IVP)) EDFs. The computational frameworks range from single-reference calculations (SV-min and Fy(IVP)), to calculations including the configuration mixing of symmetry-restored reference states (SLyMR1), with two other methods in between (BSkG2 and D1M) that include beyond-mean-field corrections to a varying degree (Methods). Figure 2 shows the experimental data and predictions on the differential mean-square charge radii δ⟨*r*^2^⟩ in fermium and nobelium isotopic chains. Figure 2a compares measured δ⟨*r*^2^⟩ values with predictions of a simple spherical droplet model^45,46^, and Fig. 2b,c compares the deviations of the experimental values and the EDF calculations from the droplet-model reference. The statistical uncertainties of charge radii stemming from the model calibration^47,48^ of Fy(IVP) and SV-min are marked. Similar uncertainty bands can be assumed for the other models.

## Discussion

As seen in Fig. 2, for the neutron-deficient even–even isotopes ^246,248,250^Fm, the experimental data agree with the predicted trend of the spherical droplet model. The heavier isotopes ^254,255,257^Fm, however, exceed the droplet-model trend. From the observed evolution of δ⟨*r*^2^⟩ in nobelium, a smooth trend consistent with the droplet-model predictions is extracted with no obvious kink at *N* = 152.

Within their uncertainties, all model predictions are strikingly consistent with the experimental data for fermium and nobelium, and with each other. These findings would suggest that in fact shell effects are smeared out in the heavy actinide nuclei^2,7^ and, hence, nuclear charge radii are expected to be primarily governed by bulk effects.

A good agreement is found between the experimental data for the neutron-deficient isotopes and most theoretical models. Larger discrepancies arise for heavier isotopes with *N* > 152. Here, Fy(IVP), owing to a generally steeper predicted incline, explains this local increase fairly well in terms of an interplay between a deformed neutron shell gap and reduced pairing. However, it is to be noted that all these small variations of the predictions are of the order of current theoretical uncertainties for charge radii.

**Fig. 3:**
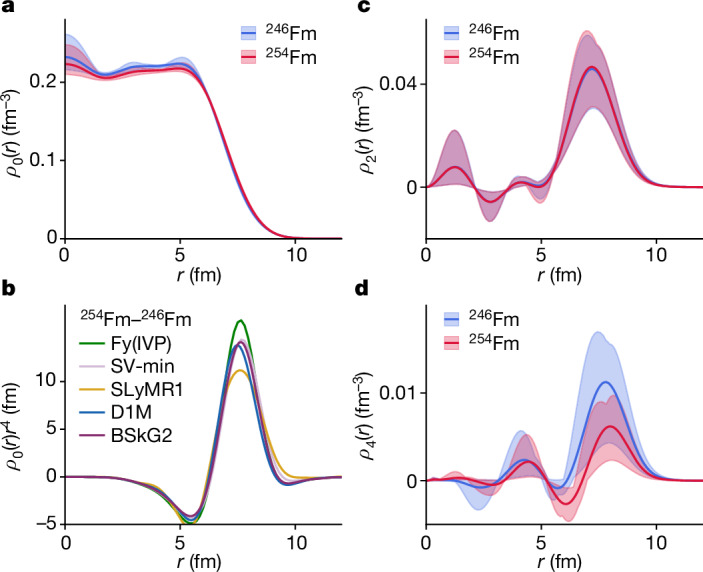
Comparison of different model predictions for multipole proton radial densities of ^246^Fm and ^254^Fm. **a**–**d**, Monopole radial densities (**a**), *r*^4^-weighted difference between monopole radial densities of ^254^Fm and ^246^Fm (**b**), quadrupole radial densities (**c**) and hexadecapole radial densities (**d**). The maximum range of model predictions is marked by bands in **a**, **c** and **d**; the solid lines represent the average of the models. Source Data

The agreement between the different predictions is highlighted in the inter-model comparison of the multipole proton radial density distributions *ρ*_*ℓ*_(*r*) calculated for ^246^Fm and ^254^Fm in Fig. 3 (see Methods for details). The radial densities *ρ*_0_ in Fig. 3a show most pronounced variations at *r* = 0, owing to slightly varying contributions from *ℓ* = 0 orbitals resulting in a small bump, a weak shell effect. For *r* > 2 fm, the radial densities steadily increase towards the surface as expected from the large Coulomb repulsion^2,33^. For radii *r* > 5 fm, which is the essential region contributing to ⟨*r*^2^⟩, the radial densities are very similar for the different models. The agreement of the weighted densities *ρ*_0_*r*^4^ between ^246^Fm and ^254^Fm in Fig. 3b reflects the similarity in predicted charge radii for these isotopes, despite their rather different neutron number. The quadrupole radial density *ρ*_2_(*r*) in Fig. 3c shows a weak model dependence. This is consistent with similar predicted quadrupole deformations along the fermium isotope chain^49^. The hexadecapole radial densities *ρ*_4_(*r*) shown in Fig. 3d show appreciable differences between ^246^Fm and ^254^Fm, which are consistent with different predicted hexadecapole deformations *β*_4_ in ^246^Fm and ^254^Fm and similar to decreasing hexadecapole moments along other actinide chains^50^.

In summary, a smooth trend in differential mean-square charge radii δ⟨*r*^2^⟩ has been observed. This is in agreement with our EDF calculations.

## Conclusion

The combination of highly sensitive laser spectroscopy techniques with multiple production schemes used in this work enabled the extraction of isotope shifts along the chain of deformed fermium isotopes and extended the isotope-shift data in the nobelium chain. The combined data on fermium and nobelium differential mean-square charge radii, interpreted by several theoretical nuclear models, suggest that the weak shell effects in this region do not influence the charge radii. This confirms theoretical expectations of the transition towards a bulk behaviour with increasing nuclear mass^2,51^.

Our results and experimental methodological advances offer good prospects for further laser spectroscopy measurements in the heaviest nuclei. The combination of the presented production schemes with anticipated measurements using the in-gas jet laser spectroscopy technique^52^, featuring a spectral resolution comparable to the resolution obtained for ^255^Fm in this work, even for isotopes accessible only on-line, will enable high-precision hyperfine structure measurements in fermium and nobelium. This will provide an experimental access to the nuclear electromagnetic moments and the spin. With respect to charge radii information for other elements and, with increased precision of experimental data, higher-order nuclear-structure effects hidden in the mean-square charge radii, such as the variations in the odd–even staggering, will be within experimental reach. Data on charge radii and on nuclear moments of heavier nuclei will be paramount to further probe the transition to the macroscopic regime and calibrate microscopic models of heavy nuclei.

## Methods

### Experimental techniques

The long chain of fermium isotopes studied in this work was measured by combining different production schemes along with two laser spectroscopy techniques for the respective measurements. Spectroscopy of the isotopes ^245,246,248,249,250,254^Fm was performed via the RADRIS technique^30,31^, with the set-up located behind the velocity filter SHIP at the GSI Helmholtzzentrum für Schwerionenforschung in Darmstadt, Germany^35,36^. Further details on RADRIS and the latest developments introduced to the set-up are given in refs. ^34,37,54^. For the on-line measurements of fermium with RADRIS, a 1 × 0.025 mm^2^ hafnium-strip filament was used for collection and neutralization of directly produced nuclei entering the buffer-gas cell filled with 95-mbar high-purity argon gas. A heat-pulse temperature of 1,450 °C was applied to desorb accumulated fermium ions from the filament as neutral atoms for subsequent laser spectroscopy. For the measurements on ^255^No, a 125*-*μm-diameter tantalum-wire filament and desorption temperatures of 1,100 °C were used.

The long-lived fermium isotopes ^255^Fm and ^257^Fm produced by neutron capture in the nuclear reactor became accessible by in-source hot-cavity laser spectroscopy at the RISIKO mass separator at Johannes Gutenberg-Universität Mainz^41,55,56^. Here, the sample was placed in a heated reservoir, with a temperature of up to 1,600 K, and the atom vapour was probed by lasers for resonant ionization. The resulting ions were extracted by an electric potential of 30 kV and mass separated using a magnetic dipole to separate the species of interest from unwanted surface ions.

#### Laser set-up for in-gas-cell laser spectroscopy at RADRIS

Laser spectroscopy of fermium was performed by exciting from the 5*f*^12^7*s*^2^ ^3^H_6_ atomic ground state to the known 5*f*^12^7*s*7*p* $\genfrac{}{}{0ex}{}{5}{}{{\rm{G}}}_{5}^{{\rm{o}}}$ atomic level around 25,111.8 cm^−1^ (refs. ^42,43^). For nobelium, the excitation occurred from the 5*f*^14^7*s*^2^ ^1^S_0_ ground state to the recently identified excited level 5*f*^14^7*s*7*p* $\genfrac{}{}{0ex}{}{1}{}{{\rm{P}}}_{1}^{{\rm{o}}}$ at ${\mathrm{29,961.457}}_{-0.007}^{+0.041}\,{{\rm{cm}}}^{-1}$ for ^254^No (ref. ^32^). A dye laser (Lambda Physics, FL series) pumped by a Xe:Cl excimer laser (Lambda Physik, LPX240) with 5-ns pulse length and 100-Hz repetition rate was used for laser spectroscopy with up to 500 μJ average energy per pulse and a spectral linewidth of 1.5 GHz using an intracavity etalon for narrow spectral linewidth operation. The laser wavelength was continuously monitored with a wavelength meter (HighFinesse-Ångstrom, WS/7-UVU) that was calibrated to an internal neon lamp. The laser light was transported to the buffer-gas cell using ultraviolet-grade optical fibres and was shaped to illuminate an area of about 3 cm^2^ around the filament. The average energy of the laser pulse at the cell was kept in a range of 70–120 μJ for the scanning laser for fermium, matching the reported saturation intensity given in ref. ^42^, and about 10 μJ for nobelium, in accordance with the measurements presented in ref. ^33^ on lighter nobelium isotopes. The pump laser for the first excitation step dye laser and the Xe:F excimer laser (Lambda Physik, LPX220), the latter providing the laser light for subsequent photoionization, were synchronized with excimer laser synchronization units (Lambda Physik, LPA 97). The ionizing laser featured about 30 mJ average energy per pulse at the cell after beam transport with mirrors. Both lasers had pulse lengths of about 18 ns.

#### Laser set-up for in-source laser spectroscopy at RISIKO

The laser system for the hot-cavity in-source laser spectroscopy of fermium isotopes at RISIKO consisted of nanosecond-pulsed titanium:sapphire lasers, pumped by two frequency-doubled neodymium-doped yttrium aluminium garnet lasers with a 10-kHz repetition rate. The titanium:sapphire lasers can be equipped with either a grating or a birefringent-etalon combination as a frequency-selective element and featured an internal second harmonic generation. One titanium:sapphire laser with an average power of up to 1.2 W was used for photoionization. A high ionization efficiency was achieved by exploiting an auto-ionizing resonance at 52,166 cm^−1^. For detailed laser spectroscopy of the first excitation step at 25,111.8 cm^−1^ in ^257^Fm, one grating-tuned titanium:sapphire laser was equipped with an additional etalon, which reduced the spectral linewidth to about 1 GHz (ref. ^57^), while the average laser power resulted in about 200 mW. Both laser beams were overlapped anti-collinearly with the ion beam in the hot cavity via a viewport at the bending magnet. For spectroscopy of ^255^Fm, the Perpendicularly-Illuminated Laser Ion Source Trap (PI-LIST) was employed using the atomic vapour effusing from the hot cavity and a perpendicular overlap of a narrow-linewidth laser to the atomic beam as discussed in more detail in ref. ^55^. Here, an injection-locked titanium:sapphire laser, seeded by a continuous-wave titanium:sapphire laser^58^ and equipped with an external single-pass second-harmonic-generation unit^59^ provided laser light with a band spectral linewidth of 20 MHz and an average power of 100 mW. A laser pulse length of 40 ns was maintained for all lasers and pulse synchronization was achieved by external triggering of the pump lasers with a pulse delay generator. The laser wavenumber of the spectroscopic transition was monitored using two commercial wavelength meters (High Finesse, WS7 and WSU), which were regularly calibrated with a laser locked to a rubidium reference cell^60^.

### Isotope production

Different production schemes were applied to access the investigated isotopes in this work for laser spectroscopy studies.

#### Direct production on-line

The isotopes ^245^Fm (*t*_1/2_ = 5.6 s) and ^246^Fm (*t*_1/2_ = 1.54 s) were produced at the velocity filter SHIP, using the fusion-evaporation reactions ^208^Pb(^40^Ar, 3*n* and 2*n*)^245,246^Fm with reported cross-sections of 32 nb for ^245^Fm (ref. ^61^) and 10 nb for ^246^Fm (ref. ^62^). An ^40^Ar^8+^ primary beam featuring a macro-pulse structure of 5 ms beam-on and 15 ms beam-off periods, with a beam energy of 185 MeV for ^246^Fm and 193 MeV for ^245^Fm, and average intensities of 2 particle microampere (1.2 × 10^13^ ions per second) was provided by the linear accelerator UNILAC. This primary beam impacted thin lead-sulfide (PbS) targets with an areal density of typically 470 μg cm^−2^ for PbS on a 30 μg cm^−2^ carbon backing and with a 10 μg cm^−2^ carbon cover layer, the latter side facing SHIP. The targets were manufactured at the GSI target laboratory and mounted on a rotating target wheel to distribute the heat from the energy loss of the primary beam over a large area.

#### Indirect production on-line

The isotopes ^248^Fm (*t*_1/2_ = 34.5 s), ^249^Fm (*t*_1/2_ = 2.6 m), ^250^Fm (*t*_1/2_ = 30 m) were obtained from the α-decay of the isotopes ^252,253,254^No, directly produced in the fusion-evaporation reactions ^206,207,208^Pb(^48^Ca, 2*n*)^252,253,254^No with respective cross-sections of 0.5 μb, 1.3 μb and 2 μb (ref. ^63^). ^254^Fm was obtained from the radioactive decay of ^254^No using the 10% electron-capture branch to ^254^Md (*t*_1/2_ = 10 m), which then decays exclusively by electron capture to ^254^Fm (*t*_1/2_ = 3.24 h). The nobelium isotope ^255^No (*t*_1/2_ = 3.52 m) was similarly obtained indirectly via the electron-capture branch (<30% (ref. ^64^), and evaluation of previous data taken at SHIP published in ref. ^65^) of ^255^Lr (*t*_1/2_ = 31.1 s).

The primary ^48^Ca^10+^ beam was delivered with average intensities of 0.8 particle microampere (5 × 10^12^ ions per second), impinging on thin ^206,207,208^PbS targets. The collection cycle of RADRIS was adapted to breed the fermium decay-daughter isotopes on the filament^34^. Accumulation was done for 25 s (^248^Fm), 295 s (^249^Fm) and 355 s (^250^Fm), before evaporation of collected atoms from the filament followed by resonance ionization laser spectroscopy. For ^254^Fm, the long lifetime of the intermediate isotope ^254^Md necessitated a collection time of 3,600 s. This indirect isotope breeding reduced the total efficiency due to decay-branching ratios, recoil implantation into the filament material, and the half-life of mother and daughter nuclide, respectively. The effective yield was especially impacted in the case of ^249^Fm, which features a similar lifetime to its mother nuclide ^253^No (*T*_1/2_ = 1.62 m) and an α-branching ratio of 55%. For the longer-lived ^254^Fm, a dedicated rotatable detection set-up consisting of three silicon detectors was used to enable longer counting times of accumulated laser ions parallel to a new collection of laser ions (see ref. ^37^).

#### Production off-line

For the production of ^257^Fm, a ^248^Cm target was irradiated in the High Flux Isotope Reactor at Oak Ridge National Laboratory, USA^66,67^. The fermium fraction of this sample, containing remaining einsteinium^40^, was first used for studies at Florida State University and then made available for Mainz University for further investigations. For production of ^255^Fm, a sample of 290 pg (1.3 × 10^14^ atoms) ^254^Es provided by Oak Ridge National Laboratory and Florida State University, USA, was encapsulated in a quartz ampule inside a titanium cylinder and shipped to the high-flux research reactor at the Institut Laue-Langevin in Grenoble, France, for a neutron irradiation of 7 days duration. After a cool-down period of 4 days and shipping to Johannes Gutenberg-Universität Mainz, Germany, the sample contained 7.5 × 10^10^ atoms of ^255^Es (*t*_1/2_ = 39.8 d) as determined by α-decay spectroscopy. This provided a generator system for the β^−^-decay daughter ^255^Fm (*t*_1/2_ = 20.1 h) present in secular equilibrium. A chemical separation of fermium was performed four times in appropriate intervals to allow ingrowth of ^255^Fm into the ^255^Es fraction between individual separations. This procedure was based on an α-hydroxyisobutyrate separation by cation exchange on a Mitsubishi CK10Y resin. The α-hydroxyisobutyrate complex was converted to a nitrate form; the final sample was obtained after cation exchange separation on an AG50WX8 column, placed on a zirconium metal foil of 10 × 10 mm^2^, which promotes the release of neutral atoms^68^, and evaporated to dryness. With this method, ^255^Fm samples of about 7 × 10^8^ atoms and one ^257^Fm sample with at most 5 × 10^7^ atoms were available for laser spectroscopy.

### Data analysis

**Extended Data Fig. 1:**
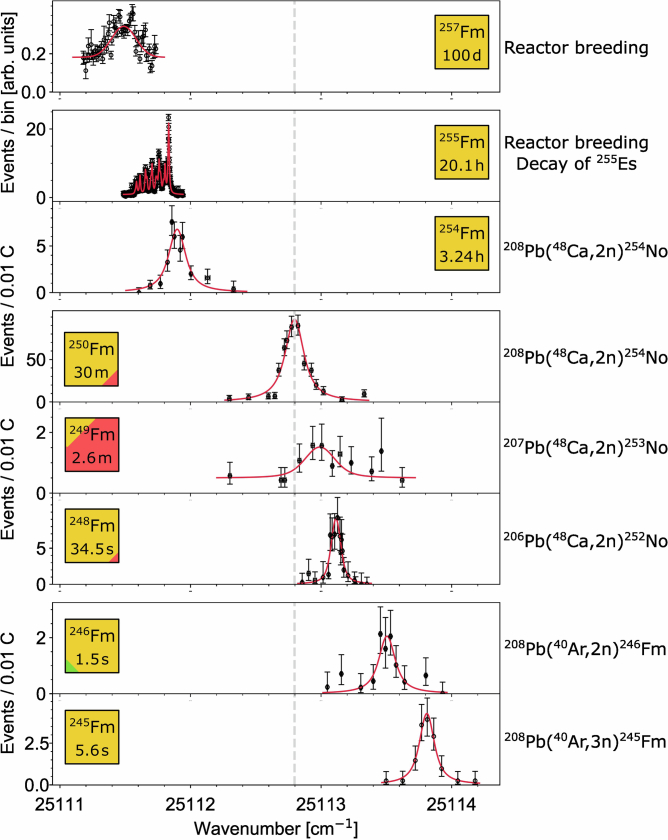
Laser resonances observed in studies of different fermium isotopes in on-line and off-line measurements. Error bars show statistical uncertainties (one standard deviation). The solid lines are fits to the data to extract the respective centroid wavenumber. The detected number of alpha events for the on-line investigated isotopes are normalized to the respective primary beam charge integral in units of 0.01 C. A more detailed zoom into the spectrum of ^255^Fm is shown as an inset in Fig. 1 in the main text. Source Data

Events from resonant laser ionization were recorded as a function of the set wavenumber to analyse the respective transition resonance centre value and thus extract the isotope shift. In the on-line measurements, the α-decay events from the ions were registered and an α-energy range of interest was selected in the analysis. To account for unavoidable fluctuations in the primary beam intensity, extracted event rates were normalized to the accumulated primary beam charge integral on the beam dump of SHIP. For the off-line measurements, the ions were detected with an ion detector after acceleration and mass separation. Gating the signal on the time-of-flight structure of the resonantly produced ions improved the signal-to-noise ratio. To average over signal variations, the laser wavenumber was scanned slowly and repetitively over the resonance multiple times and the obtained counts were binned and normalized according to the time spent at the respective wavenumber. Observed laser resonances for fermium are presented in Extended Data Fig. 1 and the resonance of ^255^No is shown in Fig. 1 (bottom).

#### Centroid position

The centroid wavenumbers of the individual measured resonances in the obtained spectra were determined via a fit of a Voigt profile to the data for all even-*A* isotopes (*A* denoting the atomic mass number). The odd-mass-number isotopes feature a hyperfine structure splitting of more than 20 lines due to (tentatively assigned) nuclear spins of *I* = 7/2 for ^249,255^Fm (refs. ^69,70^) and *I* = 9/2 for ^257^Fm (ref. ^71^), which could be only partly resolved for ^255^Fm. This spectrum was analysed using the SATLAS Python package^72,73^. Owing to the broadening mechanisms inherent in the spectral linewidth of ^245,249,257^Fm from the environmental conditions of the gas cell and the hot cavity, the hyperfine structure could not be resolved. A Gaussian fit to the data for ^257^Fm and a Voigt fit for ^245,249^Fm were used to extract the centre of the structures. The choice of fit profile was connected to the main broadening mechanisms dominating the lineshape in the respective measurement. For the analysis of ^255^No, with observed underlying hyperfine structure, a nuclear spin of *I* = 1/2 was assumed for the fit, as tentatively assigned from α-decay hindrance factor systematics^74^. The results on transition resonance wavenumbers and extracted isotope shifts are summarized in Extended Data Table 1.

The RADRIS measurements were performed inside a cell filled with 95-mbar argon buffer gas and are thus affected by a pressure shift and broadening. The broadening is effectively taken into account in the fitting routine, while the pressure shift, equivalent across all RADRIS measurements, effectively does not contribute to the isotope-shift measurement. For comparison with the off-line measurements, the pressure shift had to be evaluated. In the element erbium, a pressure shift of 4(1) MHz mbar^−1^ was recently reported^75^, which is in line with observations in actinium^76^. Therefore, a shift of about −400(300) MHz can be inferred for the in-gas-cell laser spectroscopy measurements, with a 3 times larger uncertainty assumed for the application to fermium.

The off-line measurements were performed inside the hot cavity with an anti-collinearly propagating laser beam for ^257^Fm and ^255^Fm, and with a perpendicular arrangement of the laser beam and the atomic beam for ^255^Fm. The latter measurement corresponds to the rest frame of the atom in vacuum conditions. The Doppler shift from the moving ensemble of atoms in the hot cavity can be determined from the ^255^Fm measurements (anti-collinear and perpendicular) to −100(100) MHz, in agreement with observations reported in californium^55^. For comparison with the gas-cell measurements, with ^250^Fm being the reference for isotope-shift measurements, the obtained resonance frequencies for ^255^Fm and ^257^Fm, which were measured in vacuum conditions, have therefore to be shifted by −400(300) MHz and −300(400) MHz, respectively.

The individual experimental cycle of the RADRIS technique (for details, see refs. ^30,31^) adapted to each on-line studied isotope leads to a suppression of known isomers in isotopes ^248,250^Fm, which are shorter lived than the ground state. This ensured that purely the nuclear ground state was probed.

The accuracies of the extracted centroid wavenumbers are mainly limited by broadening processes. Pressure broadening is the dominant mechanism for all measurements performed in the gas cell. For ^257^Fm, Doppler broadening owing to the high temperature in the hot-cavity environment needs to be considered. Power broadening mechanisms only had a role in the case of ^250^Fm, for which an increased laser power of 150 μJ per pulse was utilized. The origins of effects contributing to the isotope-shift uncertainty are summarized in Extended Data Table 2. This includes the accuracy in the wavenumber measurement. The fit uncertainty for extraction of the centroid position in the analysis is included. As the granularity of data points and counting statistics is small for the on-line investigated isotopes, an uncertainty of half the mean-step size in these measurements can be considered instead, to avoid underestimating the centroid uncertainty. Both factors are included in the table; the respective larger contribution was considered for the total accuracy of the isotope shift.

To account for the model uncertainty for the odd–even isotopes ^245,249,257^Fm by choosing a single line profile for the extraction of the resonance centroid, one-third of the full-width at half-maximum of the fit profile was considered in the uncertainty analysis. For ^255^Fm, the hyperfine structure was resolved, and thus the uncertainty in the determination of the hyperfine parameters was used to determine the model uncertainty in the centre of gravity.

#### Determination of δ⟨*r*^2^⟩

The changes in the mean-square charge radius δ⟨*r*^2^⟩^*A*,*A*′^ relative to a reference isotope *A* can be extracted from the measured isotope shifts via the relation

1 $${\rm{\delta }}{\nu }^{A,{A}^{{\prime} }}=\frac{{m}^{{A}^{{\prime} }}-{m}^{A}}{{m}^{{A}^{{\prime} }}{m}^{A}}M+F{\rm{\delta }}{\langle {r}^{2}\rangle }^{A,{A}^{{\prime} }},$$

with the measured isotope shift δ*ν*^*A*,*A*′^ = *ν*^*A*′^ *−* *ν*^*A*^ in the atomic transition of isotopes with mass number *A* and *A*′, the mass-shift constant *M* = *M*_NMS_ + *M*_SMS_, with normal mass shift (NMS) and specific mass shift (SMS), and the field-shift constant *F*.

Recently published results from atomic model calculations provided the field-shift constant *F* for fermium^44^ to evaluate the changes in the nuclear mean-square charge radii. This was performed analogously for nobelium in ref. ^33^, which was also used for the evaluation of ^255^No. Here, an uncertainty of 0.007 cm^−1^ of the reference isotope ^254^No in the argon buffer-gas atmosphere as stated in ref. ^32^ was assumed to contribute to the isotope-shift measurement’s uncertainty.

The error on δ⟨*r*^2^⟩^*A*,*A*′^ results from a propagation of the isotope-shift uncertainty, whereas the uncertainty from the atomic coupling factors is included as a systematic uncertainty. For the field-shift constant, which was predicted with *F* = −3.14 cm^−1^ fm^−2^, the uncertainty evaluated from the atomic calculations amounts to 10%.

Although the mass shift can be neglected for the calculation of δ⟨*r*^2^⟩^*A*,*A*′^, a contribution of the mass shift to the final uncertainty is nevertheless considered.

The NMS can be calculated to *M*_NMS_ = *m*_e_*ν* ≈ 0.4 THz × u with the transition frequency *ν* and the electron mass *m*_e_. So far, the SMS contribution can be only estimated. For *s*–*p* transitions as in our case, the SMS is usually on the order of the NMS. For transitions including orbitals with a higher main quantum number, it can be more than ten times larger. Therefore, a value of 2 THz × u, 5 times larger than the NMS, is considered as a conservative estimate for the contribution to the total systematic uncertainty in the change in mean-square charge radius^77^.

The additional effect of the isotope shift depending on the nuclear deformation proposed in ref. ^44^ was investigated. With the available information on the expected deformation change, this proposed additional effect amounts to −0.003 fm^2^, which is small compared with the uncertainties and is thus neglected.

### Nuclear-structure models

Below, we provide a brief description of the models used to interpret experimental findings. All our models are based on the nuclear EDF approach. For a detailed discussion of EDF, see refs. ^78,79^.

In this study, calculations using six different EDF models were performed: Fy(IVP) (P.-G.R. & W.N., manuscript in preparation), D1M^80^, BSkG2^81^, SV-min^82^ and SLyMR1^83^ (note that this model is called SLyMR1_3b_ in ref. ^83^). This selection aims to represent current EDF models. Concerning the functional form, SV-min and BSkG2 are based on standard Skyrme functionals and density-dependent contact pairing interactions. SLyMR1 uses an extended Skyrme functional, where the density dependences are replaced by an explicit three-body interaction^84^. D1M is based on the Gogny functional. Finally, Fy(IVP) uses the Fayans functional^85,86^. SV-min and Fy(IVP) are based on a single-reference approach, whereas D1M and BSkG2 also include approximated beyond-mean-field corrections. SLyMR1 involves explicit configuration mixing and restoration of particle-number and angular-momentum symmetries^87–89^, hence it is a multi-reference approach. The models were calibrated on experimental ground-state properties of finite nuclei but differ in the choice of the calibration data. SV-min and Fy(IVP) include a statistical analysis of the underlying parameterizations. This allows predictions to be given together with a statistical calibration^47,48^.

**Extended Data Fig. 2:**
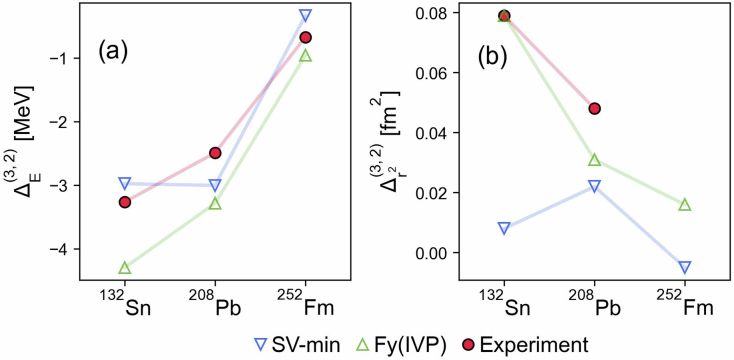
Three-point differences in the binding energy (left) and squared charge radius (right) of experimental values and calculations with SV-min and Fy(IVP). The data for binding energies were taken from ref. ^53^, for the charge radii from refs. ^24,96^. Source Data

To assess the predictive power of the theory frameworks, we computed three-point differences

$${\Delta }_{{\mathcal{O}}}^{(3,2)}=\frac{{\mathcal{O}}(Z,N+2)-2{\mathcal{O}}(Z,N)+{\mathcal{O}}(Z,N-2)}{2}$$

in the binding energy (${\mathcal{O}}=E$) or squared charge radius (${\mathcal{O}}={r}^{2}$). For *N* = 82, *N* = 126 and *N* = 152, Extended Data Fig. 2 shows predictions of the SV-min and Fy(IVP) models compared with experimental values.

Both EDF frameworks reproduce the trends in the shell gaps and agree with the data that the *N* = 152 gap is weak. As discussed in ref. ^3^, the size of this shell gap strongly depends on model details, see, for example, ref. ^18^ for the predictions of this subshell with different models. In particular, the Fy(IVP) model reproduces the experimental values for ^132^Sn and ^208^Pb, giving confidence also in the value for ^252^Fm. This is in agreement with the reported smooth trends along the isotopic chains.

#### Treatment of odd-*A* nuclei

EDF calculations for odd-mass nuclei are not straightforward: solving the self-consistent equations requires the creation of a one-quasiparticle excitation on top of a reference state that is typically associated with an even–even nucleus. For each iteration, identifying the physically relevant quasiparticle while guaranteeing convergence of the self-consistent procedure is a non-trivial task. Most calculations for odd-mass nuclei in this study concern the predicted ground state. In the case of SV-min and Fy(IVP), all one-quasiparticle configurations below 1-MeV excitation energy have been investigated, and the states with lowest energy for each angular-momentum projection *K* have been examined. It became apparent that the radii vary little with *K* (variance about 0.001 fm), such that taking the minimal-energy state is an acceptable choice. The other exception is SLyMR1: among the several many-body states with different angular momenta that result from symmetry restoration, we select those states matching the (often tentative) experimental quantum numbers. Although the blocking strategy is common to all models, they differ in their treatment of the blocked quasiparticle. D1M relies on the equal filling approximation^90^, a computationally efficient approximation that includes the blocking effect of the odd nucleon(s), but ignores the effect of any time-odd currents or densities that might develop due to the polarization effects^91^. The calculations with BSkG2, Fy(IVP), SLyMR1 and SV-min invoke no approximations in this respect.

#### Nuclear-matter properties of EDF-based models

The leading properties of our models can be characterized in terms of the infinite nuclear-matter properties shown in Extended Data Table 3: saturation density *ρ*_sat_, binding energy per particle *E*/*A*, incompressibility *K*, (isoscalar) effective mass m*/*m*, symmetry energy at saturation *J*, and slope of symmetry energy *L*. The isoscalar effective mass m*/*m* shows the largest variation. This parameter impacts the single-particle level density around the Fermi level and hence the magnitude of shell effects. The other matter parameters show fewer variations.

#### Predicted charge radii

In SV-min and Fy(IVP), the charge radii were calculated directly from the charge form factor that contains the proton form factor folded with the intrinsic form factors of the free nucleons, relativistic corrections and the centre-of-mass correction^92^. A similar procedure is followed for BSkG2 and D1M but without relativistic corrections. It is noted that D1M also adds a quadrupole correction estimated by solving the collective Schrödinger equation with the five-dimensional collective Hamiltonian to the absolute charge radius, as described in ref. ^80^. In SLyMR1 calculations, the charge radii were computed from the expectation value of the squared point-proton radius operator at the beyond-mean-field level corrected for the finite size of the protons and neutrons^88^.

To check that our models produce sensible results for the total charge radii in the heavy actinides, Extended Data Table 4 compares our predictions with the measured radii for ^232^Th, ^238^U and ^244^Pu. Given the high computational cost of multi-reference calculations, for SLyMR1, we report only the value for ^238^U. The errors given for SV-min and Fy(IVP) are the estimated extrapolation errors from statistical analysis of the underlying *χ*^2^ fits. The predictions are in good agreement with available data within uncertainties. This instills confidence in the validity of predictions for fermium and nobelium isotopes.

**Extended Data Fig. 3:**
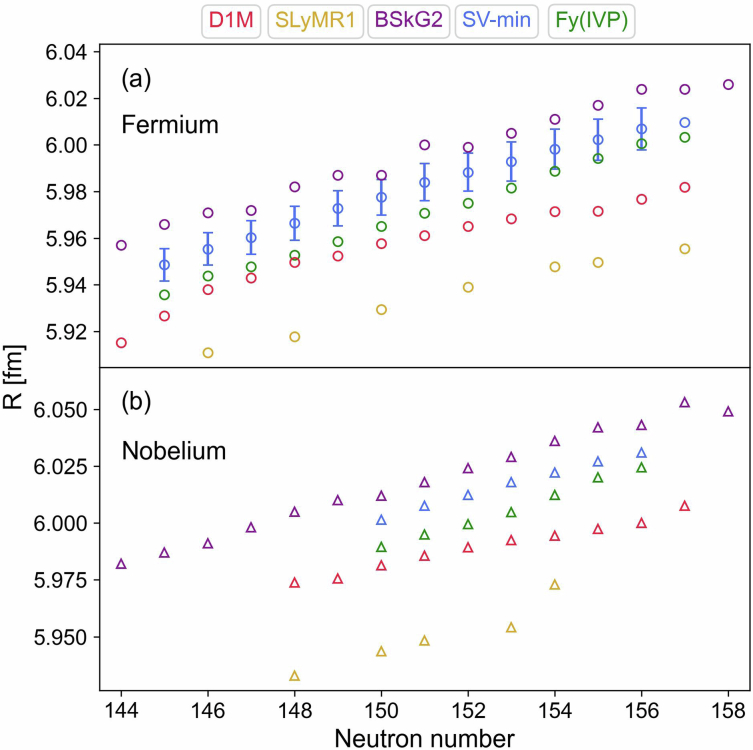
Absolute charge r.m.s. radii as predicted by the different investigated nuclear EDFs. Uncertainties (one standard deviation) for the fermium isotopes are shown for SV-min by error bars. They can be considered to be typical for most models. Source Data

The predicted root-mean-square charge radii for fermium and nobelium isotopes are shown in Extended Data Fig. 3. Prediction uncertainties are indicated for SV-min. Unlike for differential radii, the results for the total radii show a larger spread between the models. This complies with the observation that the isoscalar matter for parameters in Extended Data Table 3 differ outside error bands. Following the discussion in ref. ^93^, one would expect that the radii would be sorted according to saturation densities in Extended Data Table 3. This is not necessarily the case as the data on charge radii were used in the calibration of individual models and this spoils the correlation^93^. Still, the inter-model similarity of charge radii is related to similar saturation densities of our models.

#### Multipole decomposition of densities

The fermium isotopes under consideration are all deformed in shape, and hence their intrinsic densities are non-spherical. To make an inter-model comparison of proton densities *ρ*_p_, we carry out a multipole decomposition. To this end, we define a radial proton density *ρ*_p,*ℓm*_(*r*) as an angular average:

2 $${\rho }_{{\rm{p}},{\ell }m}(r)=\int {Y}_{{\ell }m}(\varOmega ){\rho }_{{\rm{p}}}({\bf{r}})\,{\rm{d}}\varOmega .$$

Here *Y*_*lm*_ is the spherical harmonics of degree *l* and order *m* and *Ω* represents angular coordinates. For an axially deformed nucleus, *m* = 0 and we denote *ρ*_p,*ℓ*_ ≡ *ρ*_p,*ℓm*_=0. These radial densities are related by a Fourier transformation to the radial scattering form factors typically discussed in the context of electron scattering^94^. The root-mean-square point-proton radius can be obtained from the monopole component of the proton density:

3 $$\langle {r}_{{\rm{p}}}^{2}\rangle =\frac{\sqrt{4{\rm{\pi }}}}{A}\int {\rm{d}}r\,{r}^{4}{\rho }_{{\rm{p}},00}(r),$$

where *A* is the mass number of the nucleus.

Higher-multipolarity radial densities define axial shape deformation parameters:

4 $${\beta }_{{\ell }}=4{\rm{\pi }}\frac{\langle {r}^{{\ell }}{Y}_{{\ell }0}\rangle }{3Z{R}^{{\ell }}}=4{\rm{\pi }}\frac{\int \,{\rm{d}}r\,{r}^{{\ell }+2}{\rho }_{{\rm{p}},{\ell }0}(r)}{3Z{R}^{{\ell }}},$$

where *R* = 1.2*A*^1/3^ fm. The calculated quadrupole (*ℓ* = 2) and hexadecapole (*ℓ* = 4) radial densities are shown in Fig. 3.

For the nuclei we study here, single-reference models predict nuclear densities that are deformed but retain both reflection symmetry and axial symmetry. The multi-reference techniques used in SLyMR1 render the comparison with the other models slightly more intricate. For comparison purposes, we use the multipole decomposition of the density of the deformed reference state with lowest particle-number restored energy. This deformed state also breaks axial symmetry. However, the triaxial components are small compared with the axial ones, as also found within the D1M and BSkG2 calculations.

## Online content

Any methods, additional references, Nature Portfolio reporting summaries, source data, extended data, supplementary information, acknowledgements, peer review information; details of author contributions and competing interests; and statements of data and code availability are available at 10.1038/s41586-024-08062-z.

## Source data

Source Data Fig. 1

Source Data Fig. 2

Source Data Fig. 3

Source Data Extended Data Fig. 1

Source Data Extended Data Fig. 2

Source Data Extended Data Fig. 3

## Figures and Tables

**Extended Data Table 1:**
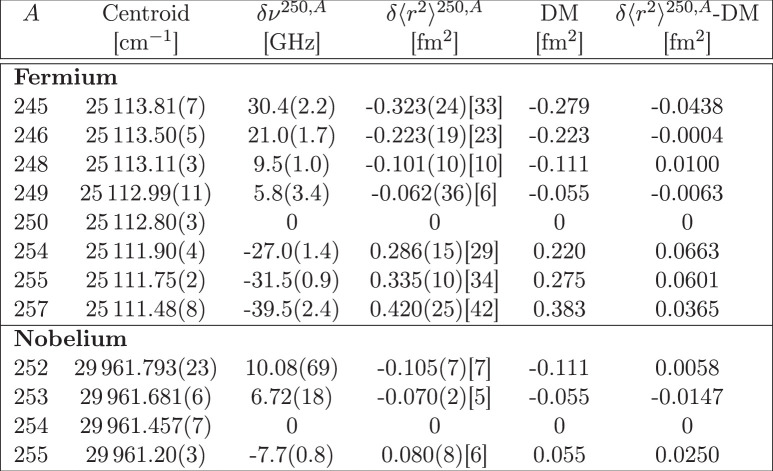
Summary of laser resonance centroid wavenumbers in argon buffer gas environment together with the corresponding isotope shifts and the evaluated changes in mean-square charge radii

Statistical uncertainties (one standard deviation) are given in parentheses, systematic uncertainties are included in brackets. Values for the isotopes ^252−254^No are taken from the literature^32,33^. The absolute values are additionally affected by the pressure shift, which cancels for the determination of the isotope shifts. Predictions by the spherical DM and relative differences to the experimental data are included.

**Extended Data Table 2:**
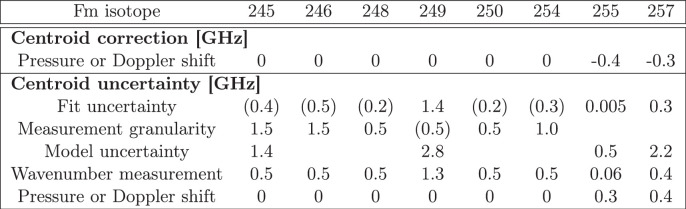
Individual contributions summed up quadratically to the total statistical uncertainty on the investigated ${\bf{5}}{{\boldsymbol{f}}}^{{\bf{12}}}{\bf{7}}{{\boldsymbol{s}}}^{{\bf{2}}}\,\,{}^{{\bf{3}}}{\bf{H}}_{{\bf{6}}}$ to ${\bf{5}}{{\boldsymbol{f}}}^{{\bf{12}}}{\bf{7}}{\boldsymbol{s}}{\bf{7}}{\boldsymbol{p}}\,\genfrac{}{}{0ex}{}{{\bf{5}}}{}{{\bf{G}}}_{{\bf{5}}}^{{\bf{o}}}$ transition for different fermium isotopes

Contributions in parentheses were not considered for the determination of the final uncertainty. For details see text.

**Extended Data Table 3:**
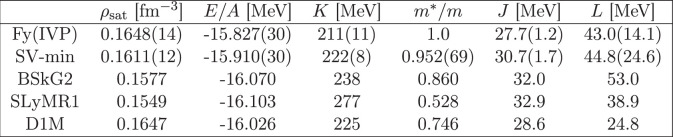
Nuclear matter properties of the EDF-based models used in this study

Uncertainties are indicated in parentheses for Fy(IVP) and SV-min. See text for details.

**Extended Data Table 4:**
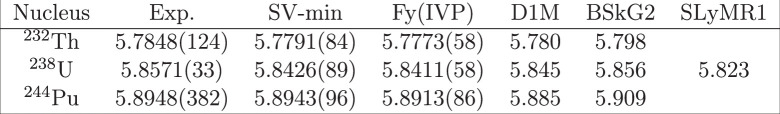
Comparison of experimental^96^ and theoretical charge rms radii (all in fm) of ^232^Th, ^238^U, and ^244^Pu

Uncertainties are given in parentheses.

## Data Availability

The data that support the findings of this study are available on Zenodo at 10.5281/zenodo.13342174 (ref. ^95^). Source data are provided with this paper.

## Code availability

The code used to analyse the data supporting the findings of this study is available on Zenodo at 10.5281/zenodo.13342174 (ref. ^95^).
